# Medium-Chain Triglycerides (MCTs) for the Symptomatic Treatment of Dementia-Related Diseases: A Systematic Review

**DOI:** 10.1155/2024/9672969

**Published:** 2024-04-12

**Authors:** Nike Meer, Tobias Fischer

**Affiliations:** FH Muenster-University of Applied Sciences, Department of Food, Nutrition, and Facilities, Corrensstraße 25, Muenster 48149, Germany

## Abstract

Pathomechanisms of dementias involve increasing damage to neuronal energy metabolism, resulting in degeneration-related insulin resistance and glucose hypometabolism. In this case, ketone bodies can provide an alternative energy source. Supplementation with medium-chain triglycerides (MCTs), which can induce ketogenesis, may alleviate brain energy deficits and improve neuronal function. This review aims to determine the effectiveness of MCT as a symptomatic treatment approach. The systematic literature search was conducted in April 2023 following the Cochrane Handbook and PRISMA guidelines. A total of 21 studies were included, comprising eight uncontrolled trials and 13 RCTs investigating the effects of MCT on Alzheimer's disease (AD) and mild cognitive impairment (MCI). A substantial increase in plasma ketone levels and brain metabolic rates was observed. Cognitive assessments showed only occasional or domain-specific performance improvements. The effects on functional abilities or psychological outcomes have been inadequately studied. Besides gastrointestinal side effects, no harmful effects were observed. However, the evidence was severely weakened by heterogeneous and poorly designed study protocols, bias, and conflicts of interest. In conclusion, the ketogenic properties of MCTs may have beneficial effects on brain metabolism in AD and MCI but do not always result in measurable clinical improvement. Current evidence is insufficient to recommend MCT as a comparable symptomatic treatment option.

## 1. Introduction

Dementia is a global problem that currently affects more than 55 million people, with nearly ten million new cases each year. The global cost of dementia and its impact on those around them, such as family caregivers, is approximately more than US$1 trillion worldwide [1, 2]. The World Health Organization (WHO) estimates that the number of people with dementia will continue to rise, and by 2030, there will be approximately 78 million people with dementia worldwide [1].

Dementias include a variety of different clinical syndromes that all involve a loss of cognitive function. The origins of dementias are diverse and can be divided into proteinopathic diseases, such as Alzheimer's disease (AD), frontotemporal dementia, and Lewy body diseases, or brain damage caused by underlying vascular diseases, like vascular dementia [3]. The most common is AD, which accounts for 60–70% of all cases. The prevalence of dementia is higher in women than in men over the age of 65 years [2], possibly due to differences in biological particularities, exposure to risk and protective factors, longer life expectancy, etc. [4]. The clinical picture of the different forms of dementia diseases depends on the location of the affected neurons [5]. In the early stages, the impairments are often subtle and can be compensated for by the person through behavioural adaptation. As the disease progresses, there are increasing disturbances in temporal-spatial orientation, communication, mobility, and ultimately often considerable impairments of everyday competence, leading to complete helplessness and even death [6, 7]. The development of dementia is influenced by many factors. In addition to the main factor of age, multiple genetic, socioeconomic, environmental, and lifestyle factors, such as diet and exercise, influence the overall risk of the disease [8].

The molecular pathological basis of neurodegenerative diseases is formed by processes of degeneration of synapses, mitochondrial dysfunction, impaired intracellular repair systems, and the accumulation of abnormal intra- and extraneuronal deposits known as plaques and neurofibrillary tangles, which consist of misfolded and aggregated proteins [9]. These changes can affect the structure and function of nerve cells, leading to impaired neuronal information transmission but also to an impaired supply of molecules and nutrients to synapses. The best known are aggregates of tau proteins, *β*-amyloids (A*β*), and *α*-synucleins, which are usually resistant to proteolytic degradation. It should be noted that the presence of such deposits is not an exclusive or specific phenomenon of neurodegenerative diseases, although they contribute significantly to the patho-neurological mechanisms in almost all of them. Plaques are also found, albeit in limited quantities, in the brains of most healthy ageing people [5, 10]. Although the formation and presence of said aggregates is still considered a major histopathological hallmark of tauopathies, ongoing research suggests that intermediate products of deposition are more likely to be responsible for disease pathogenesis than the final aggregates themselves. Prior to their formation, soluble oligomeric structures are formed, and studies suggest that these play a major role in the development of neuronal dysfunction [10, 11]. The treatment of dementia can be divided into disease-modifying and symptomatic therapies [12]. However, there is currently no cure available. The causes of many of the pathological processes involved in disease development remain unexplored and misunderstood, making research very difficult [13]. A large proportion of clinical trials for new drugs are abandoned in the early stages of testing due to significant side effects [14]. In the United States, for example, before the last two drugs (aducanumab and lecanemab) were approved by the U.S. Food and Drug Administration (FDA) in 2021 and finally in January this year [6, 15], a total of 450 clinical trials failed [13].

Due to the high prevalence and limited therapeutic options, it is not surprising that the number of studies investigating nutritional factors in dementia is increasing [14]. These include a variety of mono- and multinutrient approaches that may improve symptoms through the biochemical links between specific macro- and micronutrients and neurons. However, to date, none of these studies have been able to provide sufficient evidence to make specific dietary recommendations [8]. One potential approach involves the use of medium-chain triglycerides, or medium-chain fatty acids, which are usually defined as fatty acids with a chain length of six to ten or twelve carbon atoms. These include caproic acid (C6), caprylic acid (C8), capric acid (C10), and, under certain circumstances, lauric acid (C12) [16, 17]. The background to their therapeutic application is based on findings of considerable impairments in glucose metabolism in the brains of patients with dementia [17]. In Alzheimer's disease, *β*-amyloids are thought to damage mitochondrial electron transporters and enzyme complexes, increasing the loss of free electrons and promoting the formation of radical oxygen species (ROS), which are known to negatively affect membrane-bound glucose transporters and insulin receptors. In addition to the general age-related decrease in insulin uptake across the blood-brain barrier, this ultimately leads to the development of insulin resistance and glucose hypometabolism, which is also known as type III diabetes mellitus [18, 19]. What was initially thought to only be a by-product of synaptic dysfunction is now known to be a major driver of disease progression, as this significant energy gap can lead to neuronal cell malfunction long before clinical symptoms appear. Fortunately, this finding allows promising approaches to brain energy rescue strategies that focus on alternative metabolic pathways to improve neuronal energy supply and functionality in AD [20].

One alternative way to provide energy to the brain could be through the utilization of ketone bodies to avoid dysfunction and cell death [21, 22]. This can be achieved by a ketogenic dietary therapy, the administration of exogenous ketone esters, or the use of MCTs and medium-chain fatty acids (MCFAs) [17, 23]. Both ketones and MCFAs can cross the blood-brain barrier in a transporter-mediated manner. Ketone bodies of hepatic origin can be processed by ketolysis in brain cells for energy production, but MCFAs may also have other anaplerotic effects in addition to local ketogenesis. Neurons show a preference for ketone body metabolism, whereas astrocytes preferentially metabolize MCFAs. Differences in the metabolic effects of C8 and C10 are also evident: treatment of astrocytes with C8 primarily stimulates ketogenesis, whereas incubation with C10 leads to up to 50% increased glycolysis and the formation of lactate, the preferred energy source of neurons. This connection is also known as the astrocyte-neuron lactate shuttle (ANLS) [24]. Furthermore, research has identified other indirect ways, in which MCFAs may beneficially modulate energy metabolism in the brain, for example, by positively affecting aspects of dementia-related mitochondrial dysfunction [16]. Cell lines from AD mouse models incubated with C10 showed significant positive effects on the mitochondrial respiratory chain, enzyme activity, and an overall increase in mitochondrial number. C10 can upregulate certain metabolically relevant regulatory proteins, such as the peroxisome proliferator-activated receptors [22, 25]. By acting as a ligand for these receptors, binding of C10 leads to increased transcription of nuclear and mitochondrial genes, increasing the total number of mitochondria and thus allowing higher rates of cellular energy production [21]. In addition, C10 may also increase the enzymatic activity of the protein sirtuin 1, which also acts as a master regulator of mitochondrial biogenesis and oxidative phosphorylation [22, 26]. In terms of potential modulatory properties, it should be noted that there are significant genetically mediated differences in cellular substrate uptake and cytosolic metabolism of both glucose and ketones between different apolipoprotein E (APOE) genotypes in AD [26, 27].

Numerous studies in animal models or cell-based systems have shown positive effects of MCTs, such as anti-inflammatory and antioxidant properties, reduced amyloid secretion, or even direct amyloid degradation through stimulation of insulysin [15, 22–24]. Thus, based on the study results from in vitro and animal models and the increasing number of human studies, there is promising evidence for a potential procognitive effect of MCT, MCFAs, and their metabolites on dementia. The aim of this systematic review is to provide an overview of the current state of research on the effects of MCTs and MCFAs on dementia based on human trials.

## 2. Materials and Methods

This systematic review was conducted in accordance with the guidelines of PRISMA (Preferred Reporting Items for Systematic Reviews and Meta-Analyses) guidelines and used methodological recommendations from the Cochrane Handbook [28, 29].

### 2.1. Search Strategy

The literature search was performed from April to May 2023 on electronic bibliographic databases such as PubMed/MEDLINE (NLM), Web of Science (Clarivate Analytics), LIVIVO (ZB MED), and the Cochrane Central Register of Controlled Clinical Trials (CENTRAL) of the Cochrane Library (Wiley). The search strategy was based on a clear and careful selection of keywords and terms. Therefore, combinations of the keywords “medium-chain triglycerides,” “coconut,” and “dementia” were chosen for the literature search. The entered search strings are reported in Supplementary Material (Supplementary Table S1). The search filter was set on human studies, and the languages were restricted to English and German. The publication date was not limited to a specific period.

### 2.2. Inclusion and Exclusion Criteria

The inclusion and exclusion criteria were defined according to the PICO (Population, Intervention, Comparison, and Outcomes) scheme with the addition of study types (Table 1) [30].

### 2.3. Screening

In accordance with the Cochrane Handbook [31], the search records were exported to the literature management program Citavi (version 6.15) and merged. After automatic and manual screening for duplicates, the remaining articles were screened for relevance to the research topic and document type using the title and abstract. Two reviewers (N.M. and T.F.) independently assessed the titles and abstracts of all studies identified in the search. Publications not meeting the eligibility criteria (Table 1) were eliminated. The remaining documents were retrieved in full and, if not accessible, were requested from the authors using ResearchGate. A response time of three weeks was set for full-text requests. Registered trials were checked for publication status; unfinished or discontinued trials were excluded. Finally, the full texts were examined in detail to determine whether they met the defined criteria. Any discrepancies were discussed between the two authors.

### 2.4. Data Extraction

Data were extracted in Microsoft Excel (Microsoft 365 MSO, version 2307) according to the recommendations of the Cochrane Handbook: general information (author, title, location, year); study design (study type, randomization, blinding); participants (population, number, age, sex, drop-outs, APOE status, medication); intervention (product, dose, MCT content, control if applicable); statistical analysis methods, outcomes, and results sorted by their domain; and authors' conclusion, limitations, and conflicts of interest/funding [32].

### 2.5. Data Synthesis

Study results were categorized into (1) cognitive, functional, and psychological outcomes; (2) data on ketone body metabolism; and (3) secondary aspects of tolerability, compliance, and safety. Interventions were considered effective if there was a significant increase in plasma ketones and significant within-group, or preferably between-group, results on neuropsychological tests. In the absence of percentage data on drop-out, sex distribution, and medication adherence, a calculation was made. For missing population mean values for age and baseline cognitive characteristics, a weighted mean was calculated if appropriate information on group size was provided. Intervention dose data and outcome parameters were standardized in their units of measurement where necessary. Volumes (mL) of dose characteristics were converted to weights (g) by their densities (coconut oil = 0.92 g/mL [20°C] [33] and MCT oils = 0.95 g/mL [34, 35]). If not stated by the authors, the total MCT content and the fatty acid pattern (C8/C10) of the products used were obtained by Internet research or estimated. Plasma ketone body concentrations were uniformly converted to mmol/L.

### 2.6. Quality Assessment

Due to the heterogeneity of the study designs, different methods were used to assess the quality of the studies and the potential for bias. For controlled randomized trials (RCTs), the “Revised Cochrane risk-of-bias tool for randomized trials,” in short “RoB-II,” was used to assess the degree of bias [36]. The domains “effect of assignment to intervention” and “effect of adhering to intervention” were included in the analysis. Cohort studies were assessed using a modified version of the Newcastle‒Ottawa scale [37]. In addition, all included studies were assessed for potential conflicts of interest using a classification into three different risk levels (low = no conflict of interest; moderate = sponsored by, working with or for a company; high = additional involvement of companies in outcome collection, analysis, or publication processes). The strength of evidence was then classified using the four-level classification system of the American Academy of Neurology (AAN) Classification of Evidence framework for therapeutic intervention studies [32].

### 2.7. Data Analysis

Because of the high heterogeneity of the studies and interventions, a meta-analysis was not possible, and a narrative synthesis was performed, concentrating on the general characteristics of the included studies, participants, intervention type, study quality, evidence, and the reported effects of the interventions on dementia.

## 3. Results

### 3.1. Study Selection

**Figure 1:**
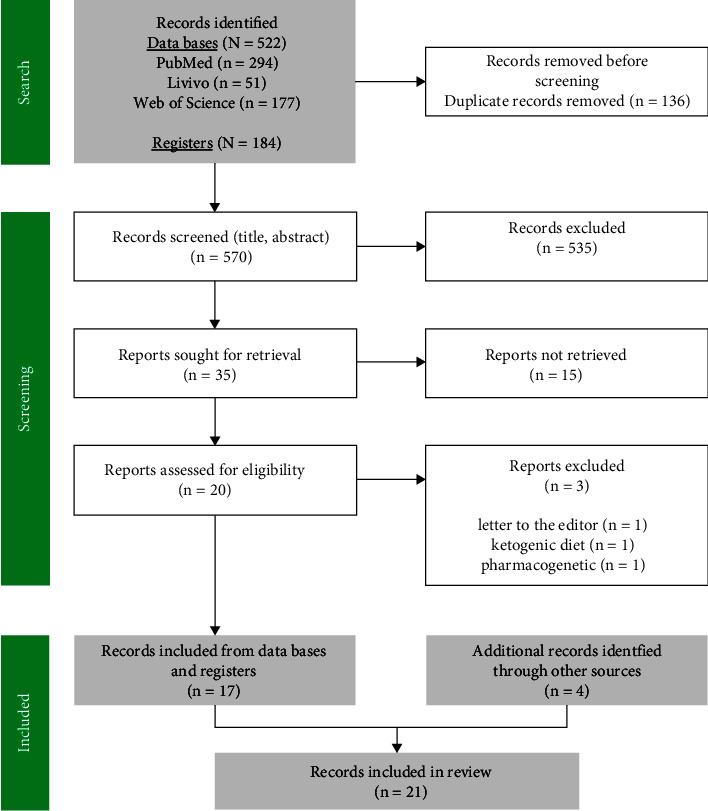
Flowchart of the literature search and screening in accordance with the PRISMA 2020 flow diagram for systematic reviews [38].

The electronic database and register search yielded a total of 570 references after removing duplicates. Title and abstract screening resulted in the exclusion of 535 records. In addition, a total of 15 articles were not retrieved (ongoing trial, foreign language, and poster abstract), and the full-text screening led to a further exclusion of three records. Reference list screening identified four additional articles, resulting in a final number of 21 publications included in this review (Figure 1).

### 3.2. Study Characteristics

The publication period of the included studies was between 2009 and 2022, with half of the studies being published within the last five years. Geographically, the majority, a total of 15 (∼70%) studies, took place in North America, followed by (South) East Asian countries. Only two studies (∼10%) were conducted in Europe. There were eight uncontrolled intervention studies, including three case studies, one retrospective cohort study, one prospective open-label study, and two pre-post study designs. All the remaining studies (*n* = 13; ∼60%) could be identified as RCTs, including two larger multicentre trials. Four of the RCTs used a crossover design.

The total number of study participants was 1193, of whom approximately 58% were female (missing data in Reger et al. [39]). The mean age ranged from 58 to 79.9 years. The number of participants varied widely, from *n* = 1 in the case studies to *n* = 413 in the largest randomized controlled trial.

The intervention duration of trials regarding chronic MCT consumption ranged from a minimum of three weeks to a maximum of six months, while the case studies reported individual cases of up to two years of use [40, 41]. The majority of studies (*n* = 13) used a dose-adaptation phase of one to two weeks to reduce MCT-associated tolerance problems. Only Ohnuma et al. and Juby et al. were the running-in period preceded by the intervention period and not part of the intervention [42, 43].

Apart from the studies of Reger et al., Rebello et al., and the four BENEFIC (Brain Energy, Functional Imaging, and Cognition) trials of Fortier et al. and Roy et al., which included mild cognitive impairment (MCI), all other studies involved people with AD. The majority of the AD diagnoses were based on the NINCDS-ADRDA (National Institute of Neurological and Communicative Diseases and Stroke/Alzheimer's Disease and Related Disorders Association), DSM-IV criteria, whereas MCI was predominantly defined by the Peterson criteria. Chan et al. did not provide a detailed description of the diagnostic procedure, but it can be assumed that the diagnostic was appropriate based on the cognitive assessment used in the study. The same is true for the studies by Farah et al., Maynard et al., Newport et al., and de la Rubia Ortí et al., which used subjects from specialized dementia centres.

At baseline, the Mini-Mental Status Exam (MMSE; 30 points = unrestricted cognition; 0 points = severely impaired cognition [44]) scores of the AD population in the studies ranged from mild to moderate severity (10.4 [45] to a maximum of 23 points [46]). Studies based on MCI showed higher scores and a much narrower range (27.2 [47]–27.5 points [48]). Overall, APOE genotyping was performed in only 16 of the included studies and mostly only in a subset of the study population. Approximately 75% of those screened were identified as APOE positive (APOE+) and had at least one of the *ε*4 alleles in their genotype.

### 3.3. Interventions and Controls

Most of the included trials included interventions based on MCT supplements (*n* = 17), including oils (*n* = 10), powders (*n* = 5), or gels (*n* = 1). The type of MCT formulation used by Ota et al. was not defined. It was usually administered as a mixture or emulsification with water or dairy products (*n* = 14). Ingestion was partly with (*n* = 7), immediately before (*n* = 1), or after meals (*n* = 3). Fasting was only used in single-dose interventions [39, 49, 50]. In eight trials, the duration of intake was not defined. Three studies included interventions containing coconut oil [45, 51, 52]. Only Newport et al. used a combination of MCT and coconut oil [41]. Almost all controlled study designs used organoleptically matched vegetable oil-based supplements as a placebo (*n* = 11). Chan et al. chose a water-containing control product, and de la Rubia Ortí and Reger et al. compared the effects of MCTs with a nonfortified baseline diet or a carrier solution (cream), respectively [39, 51, 52]. Both the content of MCT (11.7–132 g) and the fatty acid profile (C8 7.5–100%/C10 0–40%) of the products used varied widely. Eight studies used almost exclusively C8 supplements and oils, with intakes ranging from 20 to 42.8 g caprylic acid per day. The daily intake was predominantly (*n* = 11) divided into two to three portions per day. An overview of the study characteristics, interventions, and controls is shown in the Supplementary Material (Tables S2 and S3).

### 3.4. Risk-of-Bias Assessment

The risk-of-bias assessment showed substantial design-related issues in most of the randomized intervention studies. According to the RoB-II assessment, the “overall risk of bias” of almost two-thirds of the publications had to be assessed as “high risk” because of at least one high-risk domain. In the uncontrolled studies, a high risk of bias could already be assumed due to the lack of a control group. In the present RCTs, the causes were mainly found in the areas of blinding, methods of analysis to detect intervention effects, allocation, and/or adherence to the respective study group. A common problem in the assessment of study quality was insufficient explanation or nontransparency of the methods used by the authors. An overview of the results of the risk-of-bias assessment for parallel study designs is shown in Table 2, and that for crossover studies is shown in Table 3.

### 3.5. Conflicts of Interest

Only three trials (14%) had no apparent conflicts of interest with manufacturers of the intervention products (see Table 4). Very often, there was not only an involvement of paid staff and financial support but also the involvement of the companies in data collection and analysis of the results (high risk). In addition, some authors had various conflicts of interest due to company affiliations and financial benefits. In a few cases, information on patent rights was available at the time of publication, e.g., for the use of MCT oils in neurological diseases.

### 3.6. Strength of Evidence

Uncontrolled study designs were classified as AAN class IV. Only Henderson et al. [55], Xu et al., and the BENEFIC trials by Roy et al. corresponded to a class I RCT design; all others were class II/III due to too many primary outcomes, too high drop-out rates, and/or missing data on baseline characteristics.

### 3.7. Effect of Interventions

#### 3.7.1. Ketone Bodies

Ketone body concentrations were measured in 12/21 studies, with two different methods of measurement: fasting (preprandial vs. preprandial; *n* = 4) and postprandial (preprandial vs. postprandial; *n* = 10). All data were derived from laboratory plasma analyses. The postprandial increase in ß-hydroxybutyrate (ßHB) ranged from +105% to +1250% with measured concentrations of 0.250 mmol/L to 0.902 mmol/L. In studies with a control group, a significant between-group effect was consistently demonstrated (see Table 5). The same was true for all acetoacetate (AcAc) concentration data. An exception in both cases was the C8- and C10-containing interventions by Croteau et al., which showed no significant intra- or intergroup effects. When only fasting ketones were measured, no significant intra- or intergroup effect was found in any of the studies [43, 49, 54, 55].

#### 3.7.2. Cerebral Metabolic Rate (CMR)

In 4/21 studies, cerebral metabolic rates (CMRs) of ketones and glucose were measured by positron emission tomography (PET) scans and magnetic resonance imaging (MRI) [47, 56–58]. For the clustered CMR of ßHB and AcAc (CMR_ketones_) in the whole brain, Croteau et al. and Fortier et al. reported a significant increase (130–144%) from baseline in the intervention groups. All areas of the brain analysed benefited equally from the intervention, with significant results in the comparison between groups (*p* < 0.001) [56, 57]. Similarly, Roy et al. reported mean increases of approximately 116% and 122% in CMR_AcAc_ across all fascicles and brain areas studied [47]. While the C8C10 intervention of Croteau et al. was also able to produce significant positive effects in the selected areas (whole brain +180% CMR_AcAc_), the C8 intervention product was unable to produce any improvement in the cerebellum or parietal lobe, although CMR_AcAc_ in the whole brain was doubled [56]. In the follow-up study by Roy et al., significant between-group effects were also found in the dorsal frontotemporal network and in the frontal, occipital, temporal, and parietal lobes [58].

CMR_glucose_ remained unchanged in all fascicles and brain areas compared to those at baseline and in the control group [48, 56, 57]. The only exception was an 8% increase (*p*=0.039) in glucose metabolism in white brain matter in Roy et al., which was not present in the control group [58]. No differences in brain volume, cortical thickness, or cerebral blood flow were observed with the MCT intervention. The extent of neural connectivity also remained unchanged in both groups compared to baseline [56, 57].

#### 3.7.3. Mental and Cognitive Assessments

A total of 17/21 trials used tests to assess cognition. There was a high degree of heterogeneity in the choice of tests. The MMSE was most commonly used pre- and postintervention (10/21). Only one trial and two case reports found significant improvements in MMSE scores ([41, 46, 55]; see Table 6). According to Henderson et al. [55], the APOE-negative intervention group performed significantly worse than the placebo group (−0.6/+0.1 points; *p*=0.041).

Compared to the MMSE, the Alzheimer's Disease Assessment Scale-Cognitive Subscale (ADAS-Cog; scale scores between 0 and 70; higher score = more severe cognitive impairment [59]) showed more positive effects (see Table 7). Gandotra et al. reported a decrease of 4.1 points (*p* < 0.001) from baseline after only four weeks, which remained stable until week six. In the study by Xu et al., the entire intervention group also showed a significant intragroup improvement (−2.47; *p* < 0.01), but in the subanalysis, this could only be attributed to the APOE-negative (−2.62; *p* < 0.01) and not to the APOE-positive genotypes (−0.13; *p* > 0.05). The same applied to the study by Reger et al. (−1.6; *p*=0.04). Henderson et al. [54, 55] found significant effects between intervention and control groups for certain genotypes.

Other tests, such as the Montreal Cognitive Assessment (MoCA), Stroop, 16-item Free and Cued Recall (RL-RI-16), verbal fluency, trail-making test (TMT), clock-drawing test (CDT), and others, showed a similar picture with more nonsignificant than significant results or differences between the groups.

Of the 17 studies, 12 (∼70.5%) concluded that MCT intake can be considered effective in achieving procognitive alterations, although not always in all domains or subgroups investigated or with sufficient significance [39, 41, 43, 45, 46, 48–50, 52, 57, 60, 62]. In contrast, 30% (*n* = 5) stated that no positive effect can be assumed [40, 42, 51, 54, 55]. Some studies have found correlations between the concentration of ketone bodies [39, 48, 54, 57] or their metabolic rate in the brain [47, 57] and the outcomes of cognitive tests.

#### 3.7.4. Functional Assessments

Regarding the functional abilities of daily living, no significant effects could be found. In the before and after comparison, Newport et al. were able to report a significant increase in the activities of daily living (ADL) score (undefined, presumably Alzheimer's Disease Cooperative Study-ADL Scale; ADCS-ADL) of 14 points in one individual case [41], but all the other studies were unable to show any intra- or intergroup effects in the Physical Self-Maintenance Scale (PSMS), Instrumental Activities of Daily Living (IADL), or ADCS-ADL [50, 55, 62].

#### 3.7.5. Psychological Assessments

Only two studies included psychological assessments. Newport et al. reported a case-related improvement in mood and personality traits [41], whereas Chan et al. found no statistically significant effect on the Neuropsychiatric Inventory Questionnaire (NPI-Q) in the population [51].

#### 3.7.6. Quality of Life

Surveys to measure changes in quality of life were only conducted by Juby et al. and Henderson et al. [43, 55]. No changes were found in the EQ-5D-5L, whereas the patient-reported Quality of Life in Alzheimer's Disease (QoL-AD) showed a significant improvement in subjective quality of life for APOE-positive participants in the intervention group (+1.5/−0.0p, *p*=0.042). The effect was absent in the caregiver assessment and generally in the APOE-negative genotypes [43, 55].

#### 3.7.7. General Blood Parameters

Eight trials measured general blood lipids to assess the effects of MCT intervention. Basal triglyceride levels remained unchanged after the interventions [42, 43, 45, 48, 57]. Based on postprandial blood sampling, Croteau et al. reported an increase in triglycerides to 1.4 mmol/L after consumption (*p* < 0.05) [56]. Chan et al. also reported a significant but undefined increase in plasma concentrations (*p*=0.01) [51]. Total cholesterol remained largely unchanged [42, 43, 45, 48, 51, 56, 57]. Xu et al. reported a significant increase in total cholesterol to 4.66 mmol/l (*p* < 0.01) but only due to elevated HDL cholesterol levels (1.4/1.6; *p* < 0.01) [50]. Fortier et al. also showed a significant increase to 5.2 mmol/L compared to placebo (+0.4/−0.1; *p*=0.013). LDL cholesterol always remained unchanged in intra- and intergroup comparisons [43, 45, 48, 51, 56].

Fasting plasma glucose [42, 43, 47, 51, 56–58, 60], insulin levels [43, 60], kidney [42, 54], and liver parameters [42, 56] remained unchanged with a few exceptions in aspartate transaminase (AST; 22–24/21 − 20; *p*=0.03 [48]) and alanine aminotransferase (ALAT; *p*=0.027) [51]. Henderson et al. reported generally no changes in laboratory or vital parameters [54, 55].

#### 3.7.8. Safety and Tolerability

Across all included studies, a total of 243 people (20%) dropped out prematurely, of whom approximately 65% were in the intervention groups and approximately 35% were in the control groups. Ten studies reported drop-out rates between groups ranging from over 10% to as high as 60% in the intervention group [40, 48, 49, 51, 54–57, 60, 62]. When the reason for discontinuation was reported, adverse events were the most common, with gastrointestinal discomfort (diarrhoea, nausea, vomiting, and/or abdominal pain) accounting for the majority of adverse events in the intervention groups [48, 49, 51, 54–57, 62]. Difficulties in implementing the intervention (*n* = 1 [45]) or explicitly reported noncompliance [51, 54–56, 60] were rare. Gastrointestinal discomfort and withdrawal due to adverse events occurred equally in studies with and without a gradual titration phase to improve the tolerability of MCT. In the studies that used methods to measure compliance, on average between 80% and 100% of subjects were able to maintain an acceptable level of intake, usually defined as a minimum intake of 80‐90% of the scheduled dose [42, 47, 48, 54–56, 58, 60, 62]. Reasons given for poor compliance included forgetting to take the supplement, difficulties when eating out, or impracticality in everyday life [42].

## 4. Discussion

Metabolically, the ketogenic effect of MCTs was confirmed in AD and MCI patients, both when consumed chronically and after a single dose, with no difference compared to the healthy population [56]. A postprandial increase of at least twofold and up to more than tenfold of the initial ßHB level was observed in studies measuring ketone bodies. Plasma levels in the control groups remained unchanged. This significant between-group effect was also confirmed in recent meta-analyses, with a mean difference between the intervention and control groups of 0.355–0.726 mmol/L ßHB (*I*_2_ = 0%; *p*=0.02) [63, 64]. However, the plasma levels of ßHB remained below the ranges achieved with a ketogenic diet (>1 mmol/L) or administration of exogenous ketone esters (>3 mmol/L) [65–67]. When considering ketone body levels, it is important to note that the interventions varied widely, so the correlation between studies is very limited.

The extent of the correlation between MCT intake and the increase in plasma ketone body concentrations is unclear. While a linear dose-response relationship was initially suggested [68], other studies in the low-dose range (10–20 g) failed to find such a relationship [69]. Although a 20 g dose of MCTs has a greater ketogenic effect than 10 g, it does not necessarily double plasma ketones [70]. The circumstances of consumption are also important, as the ketogenic effect varies when MCTs are taken with or without a meal [71]. For example, studies suggest that taking MCTs with carbohydrates may reduce the increase in ketone body concentration [72, 73], reduce the AUC, or delay the peak plasma concentration [74]. Taking MCTs with a complex meal is unlikely to affect intestinal absorption or blood MCT levels but will slow and reduce their metabolic rate of conversion to ketones by more than 50% [75]. Overall, a sufficiently long fast before taking MCT supplements is beneficial for ketone body synthesis, and the longer the fast, the greater the ketogenic effect [76]. If a meal has already been consumed, this effect also seems to be reproducible during the day if the fasting duration is long enough [75]. A key point for the metabolic effect is also the fatty acid pattern of the intervention products. C8 has a significantly greater ketogenic effect than C10 or C12 [70]. However, contrary to this, Croteau et al. found no significant differences in plasma ketone curves between the C8 mono-product and the C8/C10 product in their study population. The authors described the observed effect as unclear and assumed that it could be explained by the disappearance of the ketogenicity of C8 over a longer period, such as a month, or that it does not occur in AD [56]. As C10 has also been suggested to play a potentially important role in enhancing neuronal energy metabolism, combined use could appear to be reasonable [22]. Fillers and excipients, e.g., in powdered MCT products, can have a negative effect on the ketogenic effect regardless of the MCT composition [55].

Another aspect influencing the ketogenic effect is administration in emulsified form, which can accelerate the rise in ketone body concentration and increase the maximum achievable level [77]. Apart from the BENEFIC trial, this only occurred in two other studies, all of which used an emulsion with dairy products [39, 47, 48, 56–58]. However, it is also important to note that dairy components such as lactose can attenuate the ketogenic effect. A further critical point in interpreting the results is that the times at which blood samples were taken to measure plasma ketones were often very different. For example, Henderson et al. were the only ones to report that blood samples were taken one hour after dosing and reported the lowest postprandial ketone body concentration (0.25 mmol/L [55]), whereas others took measurements at least two hours later [48, 54, 56, 57].

The results of the neuroimaging studies showed a clear picture of the supportive influence of MCT-induced ketone body synthesis on impaired brain metabolism. There were no differences in ketone body metabolic capacity between the diseased populations and cognitively healthy young people [78]. Based on the confirmed strong correlation between plasma ketones and the metabolic conversion rate of ketones in the brain [56, 57], an increase in the brain's metabolic rate of approximately 3–5% could be assumed in all studies based on the average plasma levels achieved [79]. Although a reduction in CMR_glucose_ generally occurs in healthy individuals on a ketogenic diet [78], it remained predominantly unchanged in the populations studied [47, 56–58]. A possible explanation for this is that in healthy individuals, glucose metabolism is proportionally replaced by ketone body metabolism, whereas, in patients with dementia, only the supply gap is compensated and ketone bodies only supplement glucose metabolism, not replace it.

The results of the neuropsychological assessments showed little evidence of positive effects on cross-domain outcome parameters such as the MMSE [46, 55] and very mixed effects in the intervention groups on the ADAS-Cog [39, 54, 55]. In the case of the ADAS-Cog, it was striking that the intervention group did not always show an improvement, whereas the control groups almost always showed a deterioration. Within the specific cognitive domains, MCT-induced improvements were observed in tests of processing speed [48, 57], attention, episodic attention [43, 48, 58], episodic and semantic memory [48, 52], temporal orientation [52], and executive function and language ability [48]. The impact of interventions on daily living, psychological development, and quality of life was examined in only a few cases and was completely unmodified, with the exception of one case report with insufficient specification of collection methods [41]. Several reasons for heterogeneity in intervention effects can be considered, such as insufficient recruitment to the study [55, 60] or even higher drop-out rates than originally anticipated in the sample calculation [48, 51, 57]. Subject characteristics at baseline may also have played a role: in isolated cases, higher initial cognitive functionality (e.g., high MMSE score), younger age [43, 62], female gender, and also lower disease severity [52] were found to be positively correlated with the procognitive effect as variables. This suggests that neuronal regeneration may be better modulated when nerve damage is not too advanced and that gender-related hormonal differences may significantly influence not only pathogenesis but also, presumably, energy metabolism [80, 81].

When considering neuropsychiatric outcomes, it is important to bear in mind that the corresponding instruments and data collection methods used may produce biased results due to a lack of blinding, subjective questioning, or learning effects. A placebo effect cannot be ruled out in dementia studies [82], especially as the blinding of subjects was not always clearly stated in the selected studies or was generally absent in open-label designs. Furthermore, it must be taken into account that a statistically significant improvement in a test score does not automatically translate into a noticeable improvement for patients or family members, and the clinical relevance of the results must be questioned. In Alzheimer's disease, a threshold of at least a four-point improvement in ADAS-Cog within six months is often used [83], which was only achieved in Gandotra et al. and almost in the dose-compliant subgroup in Henderson et al. [45, 54]. With regard to the particularities of the studies concluding that MCTs are effective in achieving specific procognitive outcomes, despite their persistent heterogeneity, the following intervention characteristics can be considered favourable to achieving effective results: chronic use, distribution over several daily doses, preprandial ingestion, and use of mixed products containing C8 and C10 MCFAs in emulsified or gel-based formulations.

Genotype has also been implicated in the effect of MCT and other interventions on dementia. APOE-negative individuals are generally considered to be more responsive to antidementia interventions such as intranasal insulin therapy [84–86]. Possible causes include genetically determined better functioning of mitochondrial enzymes in brain tissue [27] and differences in underlying insulin sensitivity [87], which may also affect ketone body metabolism. Stronger procognitive effects with lower plasma ßHB levels in APOE-negative individuals also suggest that this genotype may have a better metabolism of ketone bodies in the brain [39, 60]. However, the results of the effect of the presented MCT interventions on the genotypes were inhomogeneous. In some cases, APOE-positive individuals benefited more from the intervention [55], or no difference was found between the genotypes [42, 43, 48, 57].

Some studies have shown correlations between plasma ßHB levels and cognition [39, 48, 57] or brain ketone metabolism [47]. This was also reflected in the analysis results of Henderson et al. who were able to show stronger effects on ADAS-Cog in the analysis groups with higher cumulative doses of the per-protocol and dose-compliant population compared to the ITT, analogous to their higher plasma levels [54]. Conversely, Ohnuma et al. and Henderson et al. confirmed that the procognitive effect and the correlation were also completely absent at insufficiently elevated plasma levels [42, 55]. The above-mentioned correlations strongly suggest that the association between ketogenic diets and improved cognition is primarily due to a presumably high proportion of ketone bodies [8].

Compared with a strict ketogenic diet, which is characterized by a restrictive selection of foods that are very low in carbohydrates and high in fat and often requires regular medical monitoring [88], a temporary but comparable ketogenic effect can also be achieved by adding MCTs [69, 75]. This is particularly beneficial given that people with dementia often experience disease-related changes in food preferences, particularly toward very sweet, carbohydrate-rich foods [89], and eating abilities and habits often change drastically [90]. These specificities may be easier to address in a purely supplemental approach than in a ketogenic diet. Considering that on average, one-third of subjects took at least 10–20% less than the expected dose and still achieved significant results, intake errors or failures seem tolerable. The reason for practical difficulties such as forgetting or omitting dose units [42, 51, 60] could be that some products, such as powders, needed additional preparation steps or had to be taken in conjunction with a fixed meal pattern, which may have made it difficult to use outside from home.

Although the data do not show a consistent positive trend in cognitive parameters, at least no negative effect on cognition through MCT consumption can be demonstrated, apart from a single case in APOE-negative subjects on the MMSE [55].

Given the evidence for increased novo lipid synthesis with MCT consumption and the potentially atherogenic properties of diets high in saturated fatty acids, such as lauric acid, the primary MCFA in coconut oil, cardiovascular risk factors should also be considered in these types of interventions [91, 92]. Considering the sporadically analysed effects on other vital parameters, no health risk could be detected even with chronic use of up to six months. Furthermore, significant increases in blood lipids and liver values as well as plasma insulin occurred in the intervention groups, although these were often only short-term, postprandial increases that did not exceed clinical reference values [93, 94]. However, there are animal studies that do show potential concerning the effects of MCT on cardiac safety in healthy mice [95]. The impact of coconut oil on cardiovascular health is still controversial, with insufficient data on clinical relevance and some suggesting neutral or even beneficial effects [92, 93].

This systematic review has some limitations, mainly due to the high heterogeneity of the available studies. In addition to the relatively small number of studies included, there was a tendency for bias and conflicts of interest or strong industry funding or collaboration, which may reduce the validity of the studies. Furthermore, in addition to well-designed RCTs, other study formats were included, such as case studies, which can be classified as of low quality. The overall approach can be justified by the lack of studies. A more rigorous selection of studies would have made it difficult to reach a conclusion. The assessment of the evidence also clearly shows the shortcomings of the current study situation, which affects the preparation of this review and its validity. The heterogeneity of the available data also made it impossible to carry out a high-quality meta-analysis, which is why the format of a systematic review had to be chosen.

## 5. Conclusions

Due to the high heterogeneity, limited study quality, and existing conflicts of interest of the currently available studies, it is only possible to make a very limited statement about the symptomatic effect of MCT in dementia. Based on the available information, it can be assumed that MCT intake can significantly stimulate ketogenesis and neuronal ketone body metabolism in the brain, even at low doses, with tolerable gastrointestinal side effects in AD and MCI patients. This ketogenic effect appears to correlate with neuronal functionality but did not result in noticeable or measurable cognitive gains or clinical improvements in all cases. Overall, the current evidence is insufficient to recommend MCTs as a comparable symptomatic treatment option. High-quality trials with standardized measurements and interventions are urgently needed to fill this knowledge gap.

## Figures and Tables

**Table 1:** Inclusion and exclusion criteria of the systematic literature search.

Category	Inclusion criteria	Exclusion criteria
Population	Human, dementia diagnosed according to guidelines or accepted criteria, no or constant medication	In vitro or animal model studies, self-diagnosis, asymptomatic Alzheimer's disease

Intervention	Dietary intervention with intake or dietary enrichment with MCT- or MCFA-containing products such as special oils, supplements, formula, or also foods such as coconut, mono- and multidose approaches, specific definition of the dosage available, and constant dosage^1^	Ketogenic diets without MCT, ketone body salts, ketone body esters, change in dose, or new medication during the study

Comparison	Controlled trials (placebo, standard diet without supplementation, or standard drug therapy), uncontrolled trials	Observational studies

Outcomes	Efficacy or effectiveness in terms of cognitive, functional, and mental health using validated tools, disease progression and severity, plasma ketone body concentration, effects on metabolism, functionality, and physiology of the brain	No use of validated tools and outcomes that do not meet the inclusion criteria
	Secondary outcomes: side effects, impact on quality of life, compliance or adherence	

Types of studies	Fully completed and published studies, English or German language, no distinction made regarding the study design	Uncompleted and unpublished studies, languages other than English and German, reviews (narrative, scoping, systematic), meta-analyses, poster abstracts, in vitro or animal studies

^1^Exception: build-up phases to increase gastrointestinal tolerance.

**Table 2:** Visualization of the RoB-II tool for parallel study designs (modified from [53]).

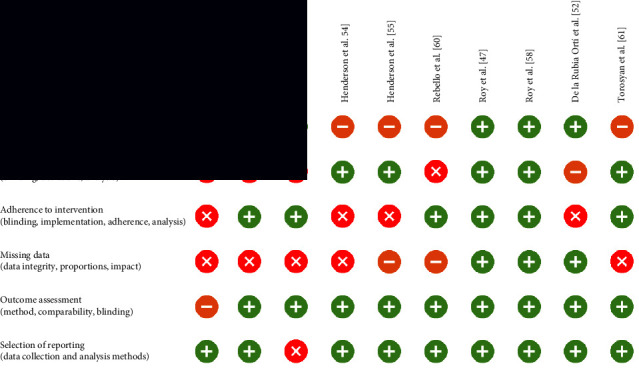

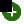
 = low risk; 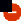 = some concerns; 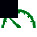 = high risk.

**Table 3:** Visualization of the RoB-II tool for crossover study designs (modified from [53]).

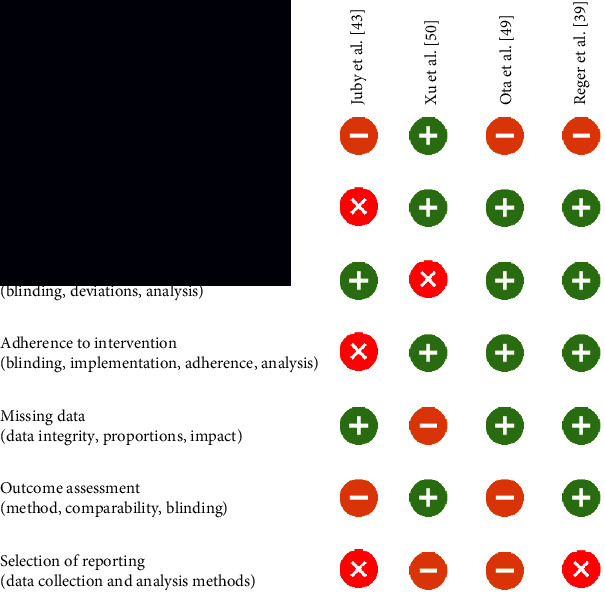

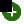
 = low risk; 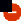 = some concerns; 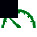 = high risk.

**Table 4:** Classification of conflicts of interest as low, moderate, and high.

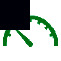	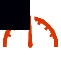	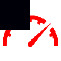
Chan et al. [51]	Croteau et al. [56]	Henderson et al. [54]
De la Rubia Ortí et al. [52]	Farah [46]	Henderson et al. [55]
Rebello et al. [60]	Fortier et al. [57]	Maynard and Gelblum [40] (case)
	Fortier et al. [48]	Maynard and Gelblum [62] (cohort)
	Gandotra and Kour [45]	
	Juby et al. [43]	
	Newport et al. [41]	
	Ohnuma et al. [42]	
	Ota et al. [49]	
	Reger et al. [39]	
	Roy et al. [47]	
	Roy et al. [58]	
	Torosyan et al. [61]	
	Xu et al. [50]	

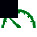
 = low; 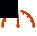 = moderate; 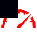 = high.

**Table 5:** Plasma concentration of *β*-hydroxybutyrate (mmol/L) in the intervention groups.

	Preintervention	Postintervention	Difference (%)	*p*
*β*-hydroxybutyrate (mmol/L)				
Preprandial vs. postprandial				
Croteau_C8_ [56]	**0.220** **±** **0.180**	**0.570** **±** **0.270**	**+159**	**0.075**
Croteau_C8C10_ [56]	0.200 ± 0.150	0.460 ± 0.190	+130	0.021
Fortier et al. [57]	0.207 ± 0.133	0.543 ± 0.321	+162	0.001^*∗∗*^
Fortier et al. [48]	0.149 ± 0.134	0.401 ± 0.303	+169	—^*∗∗*^
Henderson et al. [54]	∼0.090^*∗*^	0.390	+333	—^*∗∗*^
Henderson et al. [55]	**0.122** **±** **0.11**	**0.250** **±** **0.142**	**+105**	—
Ohnuma et al. [42]	**0.081** **±** **0.799**	**0.250** **±** **0.206**	**+209**	—
Ota_RCT_ [49]	0.065 ± 0.059	0.471 ± 0.293	+625	<0.05^*∗∗*^
Roy et al. [47]	0.210 ± 0.136	0.572 ± 0.325	+172	<0.001^*∗∗*^
Xu et al. [50]	0.393 ± 0.234	0.902 ± 0.746	+130	<0.01^*∗∗*^
Reger_*e*4−_ [39]	0.040 ± 0.020	0.540 ± 0.320	+1250	—^*∗∗*^
Reger_*e*4+_ [39]	0.080 ± 0.080	0.680 ± 0.360	+750	
Preprandial vs. preprandial				
Juby et al. [43]	**0.190**	**0.220**	**+16**	^**a**^
Henderson et al. [54]	**∼0.090** ^*∗*^	**∼0.100** ^*∗*^	**+11**	^**a†**^
Henderson et al. [55]	**0.122** **±** **0.110**	**0.128** **±** **0.100**	**+5**	—
Ota_OLE_ [49]	**0.068** **±** **0.064**	**0.106** **±** **0.157**	**+56**	**0.69**
Acetoacetate (mmol/L)				
Preprandial vs. postprandial				
Croteau_C8_ [56]	**0.140 ± 0.110**	**0.250 ± 0.070**	**+79**	**0.173**
Croteau_C8C10_ [56]	0.110 ± 0.080	0.210 ± 0.100	+91	0.014
Fortier et al. [57]	0.123 ± 0.056	0.272 ± 0.141	+121	0.001^*∗∗*^
Fortier et al. [48]	0.092 ± 0.062	0.205 ± 0.136	+123	—^*∗∗*^
Ohnuma et al. [42]	**0.035 ± 0.027**	**0.079 ± 0.049**	**+126**	—
Ota_RCT_ [49]	0.031 ± 0.022	0.138 ± 0.069	+345	<0.05^*∗∗*^
Roy et al. [47]	0.124 ± 0.054	0.286 ± 0.142	+131	<0.001^*∗∗*^
Xu et al. [50]	0.137 ± 0.067	0.257 ± 0.150	+88	<0.01^*∗∗*^
Preprandial vs. preprandial				
Ota_OLE_ [49]	**0.033 ± 0.024**	**0.043 ± 0.040**	**+30**	**0.66**

^*∗*^Estimated from figure. ^*∗∗*^Significant between-group effect; values rounded to three decimals. Nonsignificant between-group effects are shown in bold. RCT = randomized controlled trial; OLE = open-label extension; C8 = supplement containing only caprylic acid; C8C10 = supplement containing caprylic and capric acid.

**Table 6:** Intra- and intergroup effects of the MMSE.

Study	IG (pre)	IG (post)	IG difference	p (pre-post)	CG difference	IG-CG difference	p IG-CG
Chan et al. [51]	—	—	—	>0.05	—	—	>0.05
Farah [46] case report	23	28	+5.00	^a^	—	—	—
Fortier et al. [57]	27.7 ± 2.2	—	—	>0.1	—	—	—
Henderson et al. [54]	19.48 ± 4.37						
ITT	—	—	−0.206	—	−0.299	+0.09	0.8397
APOE^−^	—	—	−0.276	—	+0.385	+0.66	0.3710
APOE^+^	—	—	−0.474	—	−0.710	−0.24	0.7209
PP	—	—	−0.261	—	−0.178	+0.08	0.8925
APOE^−^	—	—	−0.056	—	+0.684	+0.74	0.4502
APOE^+^	—	—	−0.350	—	−0.913	+0.56	0.5362
DC	—	—	−0.136	—	−0.271	+0.13	0.8275
APOE^−^	—	—	−0.125	—	+0.789	+0.9	0.3656
APOE^+^	—	—	−0.136	—	−1.083	+0.95	0.2820
Henderson et al. [55]	20.8 ± 3.58						
APOE^−^	21.2 ± 3.49	∼20.6^*∗*^	−0.600	—	+0.100	+0.70	0.041
APOE^+^	19.8 ± 3.62	∼18.8^*∗*^	−1.000	—	−0.800	−0.2	0.695
Juby et al. [43]^b^							
Group 1	23.8 ± 4.7	23.4 ± 5.5	−0.4^*∗*^	0.05	—	—	—
Group 2	22.8 ± 6.4	20.1 ± 7.7	−2.7^*∗*^		—	—	—
Maynard and Gelblum [62] cohort study	20.6 ± 3.0	20.1 ± 5.6	−0.480	0.5233	—	—	—
Maynard and Gelblum [40] case report	20.3	19	−0.640^c^	—	−1.34^c^	+0.7	0.3735^c^
Newport et al. [41] case report	12	20	+8.00	—	—	—	—
Ohnuma et al. [42]					—	—	—
APOE^−^	19.1 ± 6.0	∼19.17^*∗*^	∼+0.07^*∗∗*^	>0.05	—	—	—
APOE^+^	17.8 ± 4.9	∼17.84^*∗*^	∼+0.04^*∗∗*^	>0.05	—	—	—

^*∗*^Calculated from the data. ^*∗∗*^Estimated from figure. ^a^Bayesian *p* values; before: significant deviation from norm; after: not significant. ^b^Results after extension phase 3; crossover study design with additional extension phase. ^c^Mean annual rates of decline. IG = intervention group; CG = control group; ITT = intention to treat; PP = per protocol; DC = dosage compliant; APOE−/+ = APOE positive/negative.

**Table 7:** Intra- and intergroup effects of ADAS-Cog.

Study	IG (pre)	IG (post)	IG difference	p (pre-post)	CG difference	IG-CG difference	p IG-CG
Gandotra and Kour [45]	51.3 + 14.8	47.2 + 16.3	−4.1	0.001	—	—	—
Henderson et al. [54]						
ITT	—	—	−0.313	—	+1,227	+1.54	0.0767
APOE^−^	—	—	−1.747	—	+1.614	+3.36	0.0148
APOE^+^	—	—	+0.868	—	+0.989	+0.12	0.9211
PP	—	—	−0.563	—	+0.956	+1.52	0.1923
APOE^−^	—	—	−2.426	—	+1.963	+4.39	0.0143
APOE^+^	—	—	+1.433	—	+0.145	−1.29	0.4307
DC	—	—	−1.182	—	+1.076	+2.26	0.0641
APOE^−^	—	—	−3.864	—	+1.472	+5.33	0.0063
APOE^+^	—	—	+0.909	—	+0.833	+0.08	0.9635
Henderson et al. [55]						
APOE^−^	22.1 + 9.11	23.172^*∗*^	+1.072	—	+0.311	−0.761	0.245
APOE^+^	—	—	+1.235	—	+1.217	−0.018	0.987
Newport et al. [41] case report	—	—	−6.0	—	—	—	—
Ohnuma et al. [42]	22.4 + 13.2						
APOE^−^	23.1 + 13.3	23.08^*∗*^	−0.02	>0.05	—	—	—
APOE^+^	22.7 + 14.4	22.6^*∗*^	−0.10	>0.05	—	—	—
Rebello et al. [60]						
APOE^−^	5	10	+5	—	0	5	—
APOE^+^	18	14	−4	—	−4	0	—
Reger et al. [39]						
APOE^−^	—	—	−1.6^*∗∗*^	0.04	—	—	—
APOE^+^	—	—	+1.0^*∗∗*^	>0.05	—	—	—
Xu et al. [50]	22.23 + 10.80	18.77 + 8.74	−2.47	<0.01	+2.48	−4.95	
APOE^−^	22.37 + 10.81	19.75 + 8.65	−2.62	<0.01	+2.57	−5.19	<0.01
APOE^+^	20.23 + 12.69	20.10 + 12.34	−0.13	>0.5	+1.10	−1.23	>0.5

^*∗*^Calculated from the data. ^*∗∗*^Estimated from the figure. IG = intervention group; CG = control group; ITT = intention to treat; PP = per protocol; DC = dosage compliant; APOE−/+ = APOE positive/negative.

## Data Availability

The results of the literature search can be requested from the authors. The data were processed in German.

## Supplementary Materials

Supplementary Materials Supplementary Table S1: search strategy including database, search string, and filters. Supplementary Table S2: characteristics of included uncontrolled studies; MCT = medium-chain triglycerides; m/f = male/female; CO = coconut oil; d = days; w = weeks; OLE = open-label extension; *Ø* = mean; C8 = caprylic acid; C10 = capric acid; AD = Alzheimer's disease. Supplementary Table S3: characteristics of included controlled studies; MCT = medium-chain triglycerides; LCT = long-chain triglycerides; m/f = male/female; CO = coconut oil; d = days; w = weeks; OLE = open-label extension; *Ø* = mean; C8 = caprylic acid; C10 = capric acid; AD = Alzheimer's disease; ITT = intention to treat; PP = per protocol; DC = dosage compliant.
