# Supplementary material for: Medium-Chain Triglycerides (MCTs) for the Symptomatic Treatment of Dementia-Related Diseases: A Systematic Review
Source: J Nutr Metab. 2024 Apr 12;2024:9672969. doi: 10.1155/2024/9672969 (PMC11074881; doi:10.1155/2024/9672969)
Supplement: Supplementary Materials — Supplementary Table S1: search strategy including database, search string, and filters. Supplementary Table S2: characteristics of included uncontrolled studies; MCT = medium-chain triglycerides; m/f = male/female; CO = coconut oil; d = days; w = weeks; OLE = open-label extension; Ø = mean; C8 = caprylic acid; C10 = capric acid; AD = Alzheimer's disease. Supplementary Table S3: characteristics of included controlled studies; MCT = medium-chain triglycerides; LCT = long-chain triglycerides; m/f = male/female; CO = coconut oil; d = days; w = weeks; OLE = open-label extension; Ø = mean; C8 = caprylic acid; C10 = capric acid; AD = Alzheimer's disease; ITT = intention to treat; PP = per protocol; DC = dosage compliant. [file 9672969.f1.pdf]

Medium-chain triglycerides (MCTs) for the symptomatic treatment of dementia-related diseases: A systematic review

Nike Meer [1] and Tobias Fischer [1,*]

[1] FH Muenster - University of Applied Sciences, Department of Food, Nutrition, and Facilities, Corrensstraße 25, Muenster 48149, Germany

***** Correspondence: tobias.fischer@fh-muenster.de.

Supplementary Material

Supplementary Table S1. Search strategy including database, search string, and filters.

Data base	Search	Filter
Pubmed	(medium chain OR medium-chain OR Caproic* OR Capryl* OR Capric* OR Lauric*) AND (fatty acid OR Triglyc* OR triacyl* OR acyl*OR oil) OR Tricaprilin OR Tricaprylin OR Caprylidene OR Axona OR coconut OR Palm oil OR (ketogenic AND (formula OR supplement OR agent OR Drink) OR MCFA OR MCT)<br>**AND**<br>(Dementia OR Alzheimer OR Lewy body OR Frontotemporal OR Parkinson OR MCI OR cognitive OR memory) AND (Impair* OR decline OR defect)<br>**AND**<br>(Trial OR study OR RCT OR intervention OR case)<br>**NOT**<br>(Review[Filter] OR Review[Title] OR mice[title] OR rat[title])	English, German
CENTRAL	**#1** (Medium chain OR medium-chain OR Capryl* OR Capric* OR Lau ric*) NEXT (Triglyc* OR Triacyl* OR fatty OR acid* OR acyl* OR oil*) OR MCFA OR MCT<br>**#2** Tricaprilin OR Tricaprylin OR Caprylidene OR Axona<br>**#3** Coconut* OR Palm OR Coconut Oil<br>**#4** keto* NEXT (Formula* OR Supplement* OR Agent* OR Drink*)<br>**#5** Dement* OR Alzheimer* OR ((cognit* OR memory) NEXT (Impair* OR decli* OR disorder*) OR MCI OR Lewy body OR Frontotemporal OR Par kinson<br>**#6** Dementia and cognitive Improvement".crg<br>**(#1 OR #2 OR #3 OR #4) AND (#5 OR #6)**	Trials
LIVIVO	TI=(Medium chain OR medium-chain OR Caprilydene OR Tricaprylin OR Tricaprilin OR Axona OR MCT OR MCFA OR coconut OR Palm Oil OR ketogenic Formula OR ketogenic Agent OR Ketogenic Drink OR ketogenic Supplement)<br>**AND**<br>TI=(Dement* OR Alzheimer* OR cognitive impair* OR Cognitive disorder* OR Memory Disorder* OR MCI OR Lewy Body OR Frontotemporal OR Parkinson)<br>**NOT**<br>TI=(Review OR Meta)	English, German
Web of Science	TS=(medium chain OR medium-chain OR Caproic* OR Capryl* OR Capric* OR Lauric*) AND (fatty acid OR Triglyc* OR triacyl* OR acyl) OR Tricaprilin OR Tricaprylin OR Caprylidene)<br>**AND**<br>TS=(Dement* OR Alzheimer* OR MCI OR Lewy body OR Frontotemporal OR Parkinson OR (cognit* OR memory) AND (Impair* OR decli* OR disorder*)<br>**AND**<br>LA=(English OR German)<br>**NOT**<br>TI=(Review OR Meta*)	English, German

Supplementary Table S2. Characteristics of included uncontrolled studies; MCT = medium chain triglycerides; m/f = male/female; CO = coconut oil; d = days; w = weeks; OLE = open label extension; Ø = mean; C8 = caprylic acid; C10 = capric acid; AD = Alzheimer's disease.

Author/year<br>country	Diagnosis<br>MMSE (Ø)	Age (Ø)<br>m/f (%)	APOE<br>(+/-)	N<br>(analysis)	Drop-out<br>rate	Intervention product (C8%/C10%)<br>Application form<br>Intake	Intervention duration<br>Initial phase	Dosage	g MCT/d<br>(g C8/C10)
**Case Reports**									
Newport et al., 2015<br>-	AD<br>23	58<br>100/0	100%<br>(100/0)	1 (1)	-	CO/MCT mixture (4:3)	2 years<br>35 mL CO → 165 ml MCT/CO	3-4x 165 mL	132 g<br>(68/31)[3]
Farah et al., 2014<br>USA	AD<br>23	70<br>100/0	100%<br>(0/100)	1 (1)	-	MCT-powder (C8)	109 d<br>10 → 40 g in 7d	1x 40 g	20 g<br>(20/0)
Maynard et al., 2013<br>USA	AD<br>20,3	81,3<br>75/25	12,5%<br>(100/0)	8 (8)	12,5%	MCT oil (C8)	⌀ 14,6 months[2]<br>-	1x 20 g	20<br>(20/0)
**Cohort studies**									
Maynard et al., 2013<br>USA	AD<br>20,6	79,7<br>56/44	12,7%<br>(29/71)	55 (55)	14,5%	MCT oil (C8)	⌀ 19,8 months[2]	1x 20 g	20 g<br>(20/0)
Ohnuma et al., 2016<br>Japan	AD<br>18,1	63,9<br>55/45	100%<br>(32/68)	24 (22)	8,3%	MCT powder Axona (100/0)<br>As a drink in 118-236 mL[1]<br>Postprandial[1]	90 d<br>10 → 40 g in 6d; every 2d +10 g	1x 40 g	20 g<br>(20/0)
Gandotra et al., 2014<br>Netherlands	AD<br>10,4	81,9<br>32/68	/	32 (31)	3,1%	CO native<br>Pure or mixed in<br>Postprandial for breakfast	4 weeks	1x 20 mL	11,7 g<br>(1,4/1)[3]
Croteau et al. 2018<br>Canada	AD<br>22	73,5<br>40/60	/	15 (-)	26,7%	1. MCT supplement Abitec (95/0)<br>2. MCT supplement Abitec (55/35)<br>Emulsified in 250 ml lactose-free milk<br>To the meals (2-3)	1 month each<br>4 weeks wash out<br>1 week	2x 15 g	14,25 g<br>(14,3/0)<br><br>13,5 g<br>(8,3/5,3)
Ota et al. 2019<br>Japan	AD<br>20	73,4<br>55/45	/	19 (-)	15,3%	MCT supplement Ketonformula (21,8/7)<br>As drink in hot water<br>Preprandial	12 weeks	1x 50 g	19,4 g<br>(10,9/3,5)

[1] Information from external data from the manufacturer [95]
[2] The minimum number of months of treatment is six months
[3] Estimated value based on 63,% MCT content 7,5% C8/5,7% C10

Supplementary Table S3. Characteristics of included controlled studies; MCT = medium chain triglycerides; LCT = long chain triglycerides; m/f = male/female; CO = coconut oil; d = days; w = weeks; OLE = open label extension; Ø = mean; C8 = caprylic acid; C10 = capric acid; AD = Alzheimer`s disease; ITT= intention to treat; PP = per protocol; DC = dosage compliant.

Author/year<br>country	Diagnosis<br>MMSE (Ø)	Age (Ø)<br>m/f (%)	APOE (+/-)	N (IG/KG)<br>N Analysis (IG/KG)	Drop-out rate<br>(IG/KG)	Intervention product (C8%/C10%)<br>Application form<br>Intake	Intervention duration<br>Initial phase	Dosage	Control	g MCT/d<br>(C8/C10)
**Parallel Design**										
Chan et al. 2017<br>Malaysia	AD<br>10-24	70-79<br>35/65	/	40 (20/20)<br>22 (8/14)	45%<br>(60/30)	CO native<br>Pure, mixed in food/drinks	24 weeks<br>2 weeks 2x 15 ml/d	2x 30 mL	Placebo<br>Water, coconut essence	35,1 g<br>(4,1/3,1)[3]
De la Rubia Ortí et al. 2019<br>Spain	AD<br>0-26	79,8<br>25/52	/	44 (22/22)<br>N/A	N/A	CO<br>Pure<br>With meals (morning, midday)<br>Base: Mediterranean diet	3 weeks	2x 20 mL	Mediterranean diet (15 E% protein, 55 E% complex carbohydrates, 30 E% fat, 20-30 g fiber)	23,4 g<br>(2,8/2,1)[3]
Henderson et al. 2009<br>USA	AD<br>19,6	76,9<br>48/52	88,6%<br>(56/44)	152 (86/66)<br>ITT 144 (77/63)<br>PP 91 (45/46)<br>DC 96 (46/50)	34%<br>(44/21)	MCT powder *AC-1202* (95/5)<br>(64% acacia gum, 2,6% syloid,33% MCT)<br>As drink in 230 mL liquids (milk, juice, water); Protocol change: Mixed with Abott Ensure meal replacement<br>With meals	12 weeks<br>1 week	2x 30 g	Placebo<br>(10% safflower oil, 51% acacia gum, 37% dextrose, 2% syloid); isocaloric	19,8 g<br>(18,8/1)
Henderson et al. 2020<br>USA	AD<br>20,7	76,6<br>41/59	100%<br>(31/69)	413 (208/205)<br>275 (137/138)	19,6%<br>(23/16)	MCT supplement *AC-1204* (100/0)<br>As drink in 120 mL water[1]	6 months<br>2 weeks, every 4 d +10 g	1x 40 g	Placebo<br>Sunflower oil, maltodextrin	20 g<br>(20/0)
Rebello et al. 2015<br>USA	MCI<br>25-28	58-78<br>50/50	100%<br>(50/50)	6 (3/3)<br>4 (2/2)	33%<br>(33/33)	MCT oil *Nestle*<br>In Yoplait 99% low-fat yogurt	24 weeks	1x 56 g	Placebo<br>Safflower oil	56 g<br>(39/16,8)[3]
Torosyan et al. 2018<br>USA	AD<br>21,1	79,9<br>20/70	87,5%<br>(57/43)	16 (14/2)<br>16 (14/2)	/	MCT powder *Caprylidene* (100/0)<br>As drink in 118-236 mL liquids[2]<br>Postprandial (morning, midday)	45 days	1x 40 g	Placebo<br>/	20 g<br>(20/0)
**BENEFIC Trial**										
Fortier et al. 2019<br>Canada	MCI<br>27,4	74,6<br>35/65	95% (38/62)	52 (27/25)<br>39 (19/20)	25%<br>(30/20)	MCT supplement *Captex 355* (60/40)<br>As drink emulsified in 250 mL lactose-free skim milk<br>With meals (morning, evening)	6 months<br>2 weeks 50 → 250 mL	2x 15 g	Placebo<br>Sunflower oil	30 g<br>(18/12)
Fortier et al. 2021<br>Canada	MCI<br>/	72,2<br>26/74	96% (24/76)	122 (63/58)<br>82 (39/43)	32,8%<br>(38/26)					
Roy et al. 2021<br>Canada	MCI<br>27,2	74,6<br>46/54	97% (38/62)	33 (17/16)	/					
Roy et al. 2022<br>Canada	MCI<br>27,2	75,1<br>44/66	97% (35/65)	32 (17/15)	/					

**Cross-Over Design**										
Ota et al. 2019<br>Japan<br>RCT	AD<br>20	73,4<br>55/45	/	20 (20)<br>/	-	MCT supplement *Ketonformula* (21,8/7)<br>As drink in hot water<br>Preprandial	Single dose on two separate days of visit	1x 50 g	Placebo<br>isocaloric LCT-formula	19,4 g<br>(10,9/3,5)
Juby et al. 2022<br>Canada	AD<br>22,6	72,6<br>55/45	95% (53/47)	20 (219)<br>/	5%	MCT oil *Bulletproof Brain Octane* (>99,3/0,6)	3 months each<br>1 month wash-out<br>4 weeks 15 -> 45 ml	3x 15 mL	Placebo<br>Olive oil	42,8 g<br>(42/0,3)
Reger et al. 2012<br>USA	AD, MCI<br>22	74,7<br>/	/	20 (20)<br>/	-	MCToil *Neobee 895* (95/5)<br>As drink emulsified in 152 mL cream<br>Preprandial	Single dose on two separate days of visit	1x 40 mL	Placebo<br>Cream	38 g<br>(36,1/1,9)
Xu et al 2018<br>USA	AD<br>19,2	75,1<br>37/63	100% (6/94)	53 (46)<br>/	7,5%	MCT Jelly (75/25)<br>Pure<br>Preprandial 30 minutes before meals (morning, midday, evening)	30 days each<br>30 days wash-out	3x 6 g	Placebo<br>Safflower oil jelly	17,3 (13/4,3)

[1] From trial protocol NCT02833012
[2] Information from external data from the manufacturer [49]
[3] Estimated value based on 63,5% MCT content and MCFA distribution of 7,5% C8/5,7% C10
